# Effects of somatosensory electrical stimulation on motor function and cortical oscillations

**DOI:** 10.1186/s12984-017-0323-1

**Published:** 2017-11-13

**Authors:** Adelyn P. Tu-Chan, Nikhilesh Natraj, Jason Godlove, Gary Abrams, Karunesh Ganguly

**Affiliations:** 1 Department of Neurology, University of California, San Francisco, USA; 2 Neurology & Rehabilitation Service, San Francisco VA Medical Center, 1700 Owens Street, San Francisco, California, 94158 USA

**Keywords:** Transcutaneous electric nerve stimulation, Stroke, Rehabilitation, Brain injury, Electroencephalography, Upper extremity

## Abstract

**Background:**

Few patients recover full hand dexterity after an acquired brain injury such as stroke. Repetitive somatosensory electrical stimulation (SES) is a promising method to promote recovery of hand function. However, studies using SES have largely focused on gross motor function; it remains unclear if it can modulate distal hand functions such as finger individuation.

**Objective:**

The specific goal of this study was to monitor the effects of SES on individuation as well as on cortical oscillations measured using EEG, with the additional goal of identifying neurophysiological biomarkers.

**Methods:**

Eight participants with a history of acquired brain injury and distal upper limb motor impairments received a single two-hour session of SES using transcutaneous electrical nerve stimulation. Pre- and post-intervention assessments consisted of the Action Research Arm Test (ARAT), finger fractionation, pinch force, and the modified Ashworth scale (MAS), along with resting-state EEG monitoring.

**Results:**

SES was associated with significant improvements in ARAT, MAS and finger fractionation. Moreover, SES was associated with a decrease in low frequency (0.9-4 Hz delta) ipsilesional parietomotor EEG power. Interestingly, changes in ipsilesional motor theta (4.8–7.9 Hz) and alpha (8.8–11.7 Hz) power were significantly correlated with finger fractionation improvements when using a multivariate model.

**Conclusions:**

We show the positive effects of SES on finger individuation and identify cortical oscillations that may be important electrophysiological biomarkers of individual responsiveness to SES. These biomarkers can be potential targets when customizing SES parameters to individuals with hand dexterity deficits. Trial registration: NCT03176550; retrospectively registered.

**Electronic supplementary material:**

The online version of this article (10.1186/s12984-017-0323-1) contains supplementary material, which is available to authorized users.

## Background

Despite recent advances in rehabilitation, a substantial fraction of stroke patients continue to experience persistent upper-limb deficits [1]. At best, up to 1 out of 5 patients will recover full arm function, while 50% will not recover any functional use of the affected arm. [2] Improvement in upper limb function specifically depends on sensorimotor recovery of the paretic hand [3]. Yet, there remains a lack of effective therapies readily available to the patient with acquired brain injury for recovery of hand and finger function; a systematic review found that conventional repetitive task training may not be consistently effective for the upper extremity [4]. It is thus critical to explore inexpensive and scalable approaches to restore hand and finger dexterity, reduce disability and increase participation after stroke and other acquired brain injuries.

Sensory threshold somatosensory electrical stimulation (SES) is a promising therapeutic modality for targeting hand motor recovery [5]. It is known to be a powerful tool to focally modulate sensorimotor cortices in both healthy and chronic stroke participants [5–8]. Devices such as transcutaneous nerve stimulation (TENS) units can deliver SES and are commercially available, inexpensive, low risk, and easily applied in the home setting [9]. Previous studies have demonstrated short-term and long-term improvements in hand function after SES [5, 10–15]. However, the effect of SES on regaining the ability to selectively move a given digit independently from other digits (i.e. finger fractionation) has not been investigated. Poor finger individualization is an important therapeutic target because it is commonly present even after substantial recovery and may account for chronic hand dysfunction [16]. Further, it is unclear if SES is associated with compensatory or restorative mechanisms. Prior studies have largely relied on relatively subjective clinical evaluations of impairment, such as the Fugl-Meyer Assessment, or timed and task-based assessments, such as the Jebson-Taylor Hand Function Test. Biomechanical analyses, on the other hand, can provide important objective and quantitative evidence of improvement in neurologic function and normative motor control [17, 18]. Therefore, we aimed to determine not only the functional effects, but also the kinematic effects, of SES on chronic hand dysfunction.

Simultaneously, it should be noted that although SES can potentially be an effective therapy, not all individuals who are administered SES experience positive effects. While improvement levels as high as 31–36% compared to baseline function have been reported, [11, 19] about half of one cohort demonstrated minimal or no motor performance improvement after a single session of SES [15]. One method to shed more light on this discrepancy is to identify neurophysiological biomarkers associated with motor responses to SES. Neurophysiological biomarkers are increasingly used to predict treatment effects [20, 21]. Although some studies have examined biomarkers associated with treatment-induced motor recovery, to our knowledge none have been performed for SES [22, 23]. A recent study using electroencephalography (EEG) found that changes in patterns of connectivity predicted motor recovery after stroke [24]. At present, little is known about the effect of peripheral neuromodulation on EEG activity, how existing neural dynamics interacts with peripheral stimulation, and whether this interaction is associated with improvements in motor function. Associating EEG activity with treatment response may also provide mechanistic insight regarding the effects of SES on neural plasticity. EEG activity can also potentially be used as a cost-effective real-time metric of the time-varying efficacy of SES. This novel application of EEG information may help tailor treatment efforts while reducing the variability in outcome.

The main goal of this pilot study was to evaluate both changes in finger fractionation in response to SES and identify the associated neural biomarkers through analyses of EEG dynamics. Outcomes from this study have potential in designing targeted SES therapy based on neural biomarkers to modulate and improve hand function after acquired brain injury such as stroke (e.g. enrollment in long-term studies of the efficacy of SES).

## Methods

### Ethics, consent and permissions

This research was conducted in accordance with and approval of the University of California San Francisco Institutional Review Board (IRB). All research participants provided informed consent to participate in the study.

### Inclusion/exclusion criteria

Inclusion criteria included participants between 18 and 80 years old, with a history of an acquired brain injury resulting in residual hemiparesis or other motor deficits of the arm/hand equal to or more than 6 months prior to enrollment; and capacity to adhere with the schedule of interventions and evaluations determined in the protocol. Subjects were excluded if they met any of the following criteria: currently pregnant; uncontrolled medical conditions; significant cognitive impairment on the Montreal Cognitive Assessment (MoCA ≤23); ≤ 10 degrees of active index finger range of motion; significant hand joint deformity; severe active alcohol or drug abuse; significant depression (PHQ-9 ≥ 15); baseline spasticity score (MAS) >3 for any joint tested (wrist and metacarpophalangeal joint flexion and extension); apraxia screen of Tulia (AST) <5; absent light touch, proprioception, pinprick and vibration sensation on the modified Nottingham Sensory Assessment; no upper limb strength against gravity; severe aphasia; or had an implanted pacemaker. The NSA was used for both exclusionary purposes as well as for reporting the presence of baseline sensory deficits.

Participant baseline characteristics and clinical assessments are shown in Table 1. Fourteen individuals were screened, 9 were enrolled and received the intended intervention, and 8 completed the study protocol, on which the final outcome analyses were performed. Reasons for exclusion of 5 individuals were significant cognitive impairment (MoCA <23), less than 10 degrees of active finger range of motion (two people), lack of residual motor deficits, and active treatment for brain tumor. One participant was unable to complete the study protocol due to fatigue.

**Table 1:** Summary of patient characteristics

Patient	Age	Gender	Years since injury	Affected UE	Type of Brain Injury	Lesion location	Baseline ARAT^a^	Sensory impairment	Baseline MAS	Baseline FCI^a^
1	45	F	6	Right^b^	hemorrhage	Left frontotemporal and insular lobes	49	Yes	0	0.61
2	32	M	7	Right^b^	Stroke	Left posterior frontal lobe	34	No	4	0.45
3	36	M	16	Left	hemorrhage	Right internal capsule	24	No	5	0.71
4	64	M	3	Left	Stroke	Right parietal precentral gyrus	33	Yes	1	0.86
5	72	M	1	Left^b^	Stroke	Right frontal lobe	52.67	Yes	0	0.50
6	41	M	14	Left	tumor	Left frontal lobe	33	No	5	1.51
7	66	M	6	Left	Stroke	Right posterior internal capsule and thalamus	55	Yes	0	0.42
8	28	F	2	Right^b^	TBI	Right frontal and bilateral temporal lobes, left cerrebellum	37	No	0	0.40

*TBI* traumatic brain injury, *UE* upper extremity, *ARAT* Action Research Arm Test, *MAS* Modified Ashworth Scale, *FCI*, finger coupling index

^a^Mean performance

^b^Dominant hand

### Clinical and kinematic assessments

**Fig. 1:**
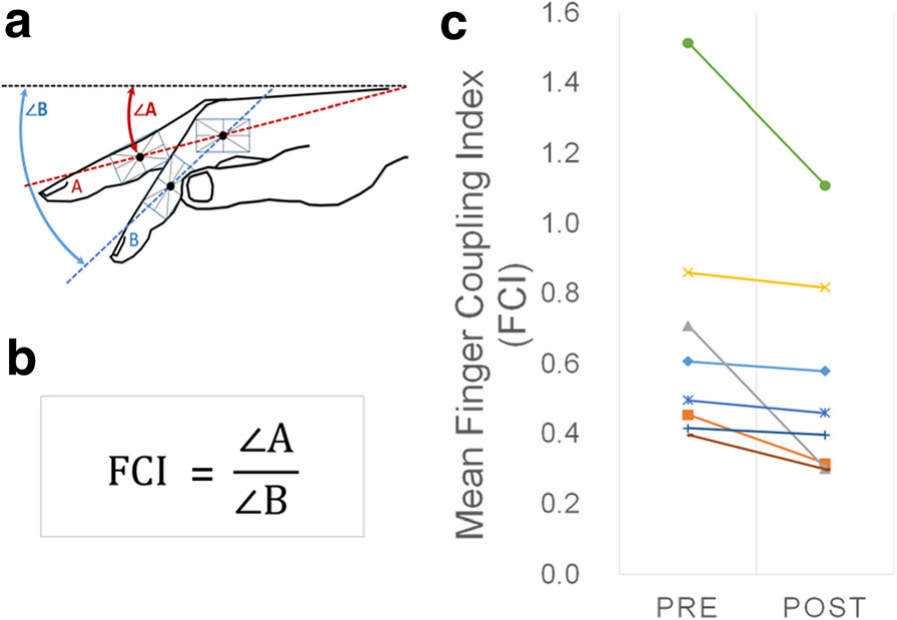
**a** Schematic representation of the method used for calculating the FCI. The participant is instructed to flex only the index finger as much as possible without flexing the other digits. **b** FCI is defined mathematically as the angle traversed by the middle finger (digit A) divided by the angle tranversed by the index finger (digit B) relative to the horizontal starting position. **c** Statistically significant change in mean fractionation from baseline to immediately after peripheral nerve stimulation. Fractionation improvement is indicated by a decrease in finger coupling index (FCI)

The primary outcome measurements consisted of the standardized Action Research Arm Test (ARAT) and a kinematic measurement of finger individuation, the finger coupling index (FCI). Participants performed multiple repetitions of the ARAT and finger individuation measurements during one familiarization session prior to the beginning of the study to address potential practice effects. The ARAT has been previously validated and was selected for its ability to measure defined domains of distal hand function (i.e. proximal, grasp, grip, and pinch tasks) [25]. Digital video recordings were obtained for kinematic motion analysis using a 30 Hz video capture system. Videos files were analyzed using a custom Matlab script to record beginning positions and end positions of the required tasks. Virtual markers were superimposed on top of recorded visual markers adhered to the participant’s hand. The beginning and end positions of each task were validated visually by video replay frame by frame. FCI was measured from frames exhibiting the maximum difference between the angle traversed by the passive middle finger divided by the angle traversed by the active index finger. (Fig. 1a-b). Three trials were averaged to obtain the mean finger coupling index. Given frequent rest breaks, participants did not have any difficulty completing the required number of trials per task. Trials that were interrupted or failed due to technical errors were discarded, and an additional set of trials would be repeated from the beginning. Secondary outcome measurements included finger pinch force (standardized dynamometer), and the Modified Ashworth Scale (MAS) to assess spasticity affecting wrist and finger flexion and extension. Outcome assessments were measured immediately before and after the intervention. Participants wore an EEG cap (Enobio, Neuroelectrics Corp., Barcelona, Spain) consisting of pre-determined electrode positions located anatomically according to the International 10–20 EEG System. Resting state EEG data with eyes open was acquired (Enobio, Neuroelectrics Corp., Barcelona, Spain) for a duration of 10 min before and after stimulation, using 8 electrodes over the Fp1, Fp2, C3, C4, P3, P4, O1, O2 at 500 Hz with a mastoid reference. Kinematic and functional outcome measurements were performed without blinding. Participants were aware of the research question regarding whether somatosensory electrical stimulation had any effect on hand motor function.

### Intervention

TENS was performed using a commercially available device (ProStim, Alimed Inc., Dedham, Massachusetts, USA). One pair of 2 × 3.5 in. rectangular electrodes (Vermed ChroniCare TENS Electrodes, Vermed, Buffalo, NY, USA) were placed on one aspect of the forearm to simultaneously stimulate both median and ulnar nerves, while a second pair of round 2 in. diameter electrodes were placed on the lateral aspect of the forearm to stimulate the radial nerve. (Additional file 1: Figure S2) Optimal positions to stimulate the ulnar, median and radial nerves of the paretic hand were determined by using standard localization technique [26, 25]. Sensory thresholds (minimum intensity of stimulation) at which subjects report paresthesias in each nerve territory were determined. Stimulus intensity was further increased and adjusted until subjects reported strong paresthesias in the absence of pain and visible muscle contractions. The mean stimulation intensity was 5.3 mA (19% above mean sensory threshold) for the radial nerve and 5.8 mA (29% above mean sensory threshold) for the median/ulnar nerves. Bursts of electrical stimulation at 10 Hz (100 microsecond pulse width duration) were delivered to all nerves simultaneously for 2 h [5, 10, 12–15, 18]. During the stimulation period, the affected hand was at rest while participants read or viewed a film.

### Statistical analyses

Experimental data were collected immediately before and after the intervention. Intervention effects were determined using non-parametric bootstrap tests to assess the difference between the pre- and post-intervention means [26]. Statistical significance was set at *p* < 0.05. Continuous 10 min EEG resting state data were epoched into non-overlapping 1000 ms time-voltage data segments and mean-baselined, with the “right hemisphere” as the common lesion hemisphere. In essence, this involved flipping hemispheric cortical activity for left hemispheric patients. Artifact correction on the epoched data was performed using a combination of principle component analysis (PCA) and the 3 S.D. voltage metric [27] to reject epochs that had abnormally large voltage values due to eye blinks, head-motion or extraneous noise. Bilateral sensorimotor electrodes (C3-C4 and P3-P4) formed the regions of interest. Resting state power was computed within each epoch across four frequency bins (delta 0.9–3.9 Hz, theta 4.8–7.9 Hz, alpha 8.8–11.7 Hz, beta 12.7–30.27 Hz) via averaging the absolute values of short time Fourier transforms (STFT) on non-overlapping 256 ms snippets within each epoch. Subsequently, the percentage change in mean resting state power, pre to post intervention, was computed for each subject at each frequency bin and electrode. A bootstrap test was used to assess the null hypothesis of group-level changes in mean resting state power being similar to a distribution with mean zero. The percentage change in mean FCI was regressed onto the percentage change in mean resting state power across all 4 bins and 4 electrodes via multivariate regression. Given that there were more predictors (changes across 4 channels X 4 frequency bins = 16 predictors) than measurements (changes in 8 subjects’ FCI), penalized regression was performed to counter effects of multicollinearity. Specifically, we used ridge regression and the ridge parameter was identified via leave-one-out cross validation [28]. It should be noted that both simple and penalized regression is susceptible to outliers given that the objective functional to be minimized is quadratic (least squares error minimization). Given the low sample size of 8 subjects, rather than reject data we used robust multivariate regression that automatically corrects for outliers based on a function of the least squares error. Specifically, robustness was implemented via an iterative re-weighted least squares algorithm based on Huber’s weighting function [29]. A permutation test was used to determine significance of the ridge coefficients that are associated with changes in mean resting state power with changes in mean FCI [30]. Bonferroni corrections for multiple comparisons were performed wherever appropriate.

## Results

Results of kinematic and clinical outcome measurements are presented in Table 2. Mean scores were significantly improved after peripheral nerve stimulation for measures including ARAT total score, overall ARAT completion time, ARAT pinch tasks subset completion time, finger coupling index, and MAS. The mean change in ARAT score was 1.5 points change (or 3.75% improvement) after one session of SES (*p* < 0.05). ARAT domain subsets were further analyzed to determine whether one specific domain improved or a generalized effect in distal upper limb function could be observed. Significant improvement was noted for speed (overall time to complete all tasks decreased by 1.72 s (13.31% change; *p* < 0.05) and pinch tasks time which reduced by 7.26 s (29% change; p < 0.05). Changes in proximal tasks time, grasp tasks time, and grip tasks time were not significant. Finger fractionation significantly improved; FCI decreased from 0.68 to 0.53 (22% change). Of the secondary outcome measurements, MAS decreased significantly by 1.13 points (60% change) amongst those who had baseline spasticity (*p* < 0.05), while mean pinch force increased by 1.22 pounds of force (11.3% change).

**Table 2:** Results of kinematic and functional outcome measures (Mean)

	Pre	Post	Absolute change	% Change	*P*-value
ARAT					
Total Score (57 points max)	40	41.5	1.5	3.75	0.008
Overall Time (sec)	12.92	11.2	−1.72	−13.31	0.004
Proximal Task Time (sec)	1.29	1.25	−0.04	−3.9	0.823
Grasp Tasks Time (sec)	6.28	6.75	0.47	7.46	0.361
Grip Tasks Time (sec)	8.3	11.3	3.03	36.4	0.058
Pinch Tasks Time (sec)	25.04	17.78	−7.26	−29	0.002
Pinch Force (lb)	10.8	12.03	1.22	11.3	0.048
MAS (16 points max)	1.88	0.75	−1.13	−60	0.010
Finger Coupling Index	0.68	0.53	−0.15	−21.63	0.006
Active Range of Motion (degrees)	68.5	75.3	6.84	9.98	0.001

*ARAT* Action Research Arm Test, *MAS* Modified Ashworth Scale, *Pre* pre-intervention performance, *Post* post-intervention performance

**Fig. 2:**
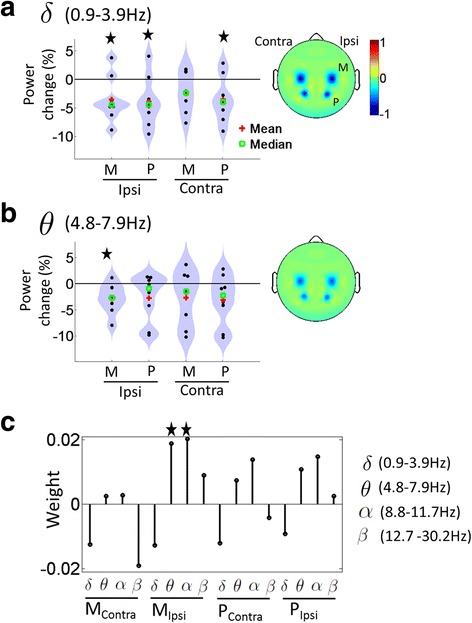
Distribution of percentage change in mean resting state EEG power across the eight subjects, pre to post intervention, within the (**a**) delta frequency band and (**b**) theta frequency band with head plots depicting 1/coefficient of variation (mean/standard deviation) of group level percentage changes. Star sign represents a significant change in group level resting state EEG power from zero. **c** Magnitude of the coefficients of the multivariate robust ridge model from regressing mean FCI changes to mean power changes, pre to post intervention, with the star sign depicting coefficients whose absolute magnitude were greater than 95% of those produced by random data permutation. M: electrodes over Motor cortex; P: electrodes over Parietal cortex

Results of resting state EEG analyses are shown in Fig. 2. At the group level, stimulation caused significant decreases primarily in mean ipsilesional resting state power at low frequencies (delta 0.9–3.9 Hz and theta 4.8–7.9 Hz bands, *p* < 0.05, Bonferroni corrected, Fig. 2a-b). In contrast, no significant changes were found for alpha and beta frequencies (Additional file 2: Figure S1A, B). In addition, combined theta and alpha power changes over the ipsilesional motor cortex were significantly correlated with fractionation changes (p < 0.05) when controlling for all other predictors in the multivariate robust ridge regression model (Fig. 2c). The ridge parameter value of 12.13 was obtained via leave one out cross-validation (Additional file 2: Figure S1C, D) and visual assessment of the quantile-quantile plot from the regression showed normally distributed residuals (Additional file 2: Fig. S1E). It should be noted that ridge regression shares coefficient values amongst correlated predictors (theta and alpha are closely related frequencies) while shrinking coefficients of predictors not correlated with the response variable.

## Discussion

Our primary results showed that a single two-hour session of SES resulted in statistically significant improvements in functional measurements as well as finger kinematics in individuals with chronic acquired brain injury. Improvements were found in the domains of activity (i.e. ARAT) and impairment (i.e. pinch strength, spasticity, and finger fractionation). A statistically significant improvement was detected in the mean ARAT score after only one session of SES. This finding is broadly consistent with similar studies of the effects of SES on hand function in stroke patients [3, 5, 15, 19, 31]. One particular study using the ARAT, however, did not find any change after SES. It was determined to be largely due to a ceiling effect [12]. For example, their participants averaged a higher baseline ARAT score than the participants in the present study. While the change in ARAT score was small in magnitude, it may be of clinical relevance; larger or additive effects have been demonstrated with multiple stimulation sessions and in combination with motor training [32, 33].

The relationship between SES and recovery of individuated finger movements has not been investigated in previous studies. Past studies mainly focused on functional measurements as outcomes, such as the Jebson-Taylor Hand Function Test, or on relatively subjective evaluations of impairment, such as the Fugl-Meyer Assessment, to determine the efficacy of SES [5, 10, 15, 19, 31]. Combining functional clinical evaluations with kinematic measurements of finger fractionation is one strategy we implemented to distinguish between functional improvements solely related to compensatory changes versus recovery of impairments. For the purpose of this study, we defined treatment-induced motor recovery as a relative improvement in finger fractionation ability after peripheral nerve stimulation. Our finding here of normalized finger fractionation kinematics suggests that SES can modulate the neural control of finger dexterity. This observation is consistent with a prior study demonstrating immediate improvement in index finger and hand tapping frequency after a single 2-h session of SES. [13] Interestingly, the ARAT total score improvement was specifically attributable to improved performance in pinch tasks rather than performance of grip, grasp, or proximal tasks. This indicates that SES may have a highly specific or greater effect on tasks that require relatively more finger individuation. However, findings of improvements in peak velocity of the wrist during reach-to-grasp tasks after SES have also been reported. [13] Although the differential effect of SES on the various aspects of upper limb function needs further evaluation, the findings taken together underline the importance of emphasizing recovery of finger dexterity to facilitate meaningful and measurable functional improvements.

The specific mechanism for increased fractionation ability after SES is unclear. Prior research suggests that SES affects complex motor skill performance by re-organization and altered excitability of the sensorimotor cortex. Neuroanatomical, electrophysiological, and imaging data revealed that unilateral electrical stimulation, including SES, can activate the contralateral S1 and S2 bilaterally [34–38]. Direct connections between Brodmann areas 1 and 2 of S1 and M1, and S2 and M1 could provide a neuroanatomical basis for the observed effects [39–43]. Furthermore, when patients with pure motor lacunar strokes have interrupted corticospinal projections at a subcortical location, the remaining descending pathways mediating voluntary movement are unable to produce selective patterns of muscle activation required for finger individuation tasks. [16] This underlines the importance of motor cortex output for the orchestration of individuated finger movements. Studies have shown no effects on peripheral nerve M-wave and spinal cord excitability (H waves) with SES, further suggesting that the changes in excitability most likely occur at the level of the cortex. [44, 45]

It has been proposed that finger individuation is a result of not only the voluntary movement of one digit but also the inhibition of digits intended to remain stationary [16]. One study using high frequency SES found a reduction in motor evoked potential (MEP) from the muscle stimulated and an increased MEP from the antagonist muscle [45]. A more recent study found increased MEP with supramotor threshold stimulation and reduced MEPs with SES [44]. Although these results cannot be directly compared to our findings because the stimulation parameters and conditions were dissimilar, they illustrate the complexity of the parameter-dependent effects of SES that can be both facilitatory as well as inhibitory. Therefore, it is plausible that SES improves motor control during finger individuation tasks by modulating cortical excitability and inhibiting inappropriate antagonist and agonist muscle co-contractions, a hypothesis in need of further exploration. The plausible neural correlates underlying the proposed corticomotor excitability changes are addressed in the following paragraph based on our EEG results.

The EEG results suggest that the observed improvements in motor kinematics and function after SES may be primarily related to changes to ipsilesional cortical oscillations. There were two results detailing the neural plasticity induced by SES that are suggestive of the aforementioned link. First, we observed a relative decrease in ipsilesional resting state low frequency power primarily in the delta band (and ipsilesional motor theta band) immediately after SES when compared to the baseline resting period. Secondarily, a decrease in ipsilesional motor theta and alpha power (two closely coupled frequency bands that were pooled together in the multivariate ridge regression model) were significantly correlated with fractionation changes with SES. Together, our results highlight the importance of reductions in low-frequency, ipsilesional cortical oscillations in association with improved behavioral responses to SES. It is thought that the loss of functional outputs from injured or damaged neurons in affected brain regions [46, 47] can result in an increased synchronous ‘idling’ state [48] of the cortical pathways as a whole. The increased idling is recorded at the surface EEG as a pathological increase in low frequency power [49]. A potential reason as to why lower frequency oscillations in particular are affected could be due to the slow oscillatory nature of blood flow and metabolism in neuronal tissue. [46, 50] In any case, an increase in low frequency ipsilesional oscillations can be thought to correspond to increased inhibitory activity in the underlying neural tissue [49]. Indeed, a recent study suggested that the reduction of resting-state low-frequency cortical oscillations are a predictor of spontaneous recovery [51]. Here, we show that SES lowers the aforementioned ipsilesional low-frequency oscillations with correlated improvements in behavioral outcomes. Mechanistically, SES could therefore serve to induce cortical plasticity in ipsilesional neural tissue by transitioning the affected region from a synchronous idling state to motor-function related activation. [48, 52]. While the low frequency power changes observed here resulted in better motor behavior, further work (e.g. corticomuscular coherence) is necessary to understand how these power changes relate to individual components of agonist and antagonist muscle activity underlying finger fractionation. Overall, our data provide evidence that neuromodulatory approaches that further reduce low frequency oscillations may be critical to improving motor function. This finding is broadly in line with changes observed in low frequency dynamics during recovery from stroke in a rodent model [53].

Our study also demonstrates how EEG features can be used as biomarkers of SES-induced recovery. In general, EEG has been correlated with motor skill acquisition in healthy individuals and as a biomarker of motor system function and improvements with physical interventions in stroke patients [23]. EEG is a safe, inexpensive, and wearable technology with the potential not only for objectively stratifying candidates, but also for serving as a biomarker of responsiveness to treatment in the outpatient setting. These preliminary findings warrant further exploration to advance our ability to select appropriate candidates for longer-term studies of SES and to customize rehabilitative treatments to individuals.

In summary, we demonstrated the feasibility of using a wearable EEG system with 8 channels to monitor and serve as a biomarker of treatment response. However, using a higher resolution EEG system with a greater number of channels may be more informative, albeit more cumbersome to apply. Given the small sample size, it is unclear whether inhomogeneity of baseline sensory impairments would impact individual responses to SES. Investigations into the impact of sensory deficits and generalizability of findings in a larger patient population is warranted. Future studies will also need to address other potential limitations of this pilot study, including the need for a randomized, controlled study design, monitoring of long-term effects of SES, varying dosing and stimulation parameters to determine their effects on EEG, and explorations into the mechanisms for the effects of SES on complex motor skills.

## Conclusions

A single 2-h session of SES can improve finger fractionation and hand function in participants with chronic acquired brain injuries. We also identified cortical oscillations using EEG that may be important electrophysiological biomarkers of individual responsiveness to SES. These biomarkers can be potential targets when customizing SES parameters to optimize its effects on individuals with residual hand dexterity deficits.

## Additional files

Additional file 1: Figure S2. (A) Placement of the rectangular electrodes overlapping the stimulation sites of the median and ulnar nerves. (B) Placement of the circular electrodes over the stimulation site of the radial nerve. (TIFF 846 kb)

Additional file 2: Figure S1. Distribution of percentage change in mean resting state EEG power across the eight subjects, pre to post intervention, within the A) alpha frequency band and B) beta frequency band with head plots depicting 1/coefficient of variation (mean/standard deviation) of group level percentage changes. There were no significant differences. C) Result from the leave-one-out cross validation (CV) procedure to find the optimal ridge parameter (lambda) that produced the lowest CV error given by the vertical dotted red line. D) Ridge trace plotting the coefficient weights of the multivariate ridge model for various values of the ridge parameter with the optimal lambda indicated by the dotted red line. E) Quantile plots from the weighted residuals of the Huber robust regression. M: electrodes over Motor cortex; P: electrodes over Parietal cortex. (TIFF 362 kb)

## Abbreviations

ARAT: Action research arm test
AST: Apraxia screen of Tulia
EEG: Electroencephalography
FCI: Finger coupling index
MAS: Modified ashworth scale
MoCA: Montreal cognitive assessment
PCA: Principle component analysis
SES: Somatosensory electrical stimulation
STFT: Short time fourier transforms
TENS: Transcutaneous nerve stimulation
