# Supplementary figures and images for: Effects of somatosensory electrical stimulation on motor function and cortical oscillations
Source: J Neuroeng Rehabil. 2017 Nov 13;14:113. doi: 10.1186/s12984-017-0323-1 (PMC5683582; doi:10.1186/s12984-017-0323-1)

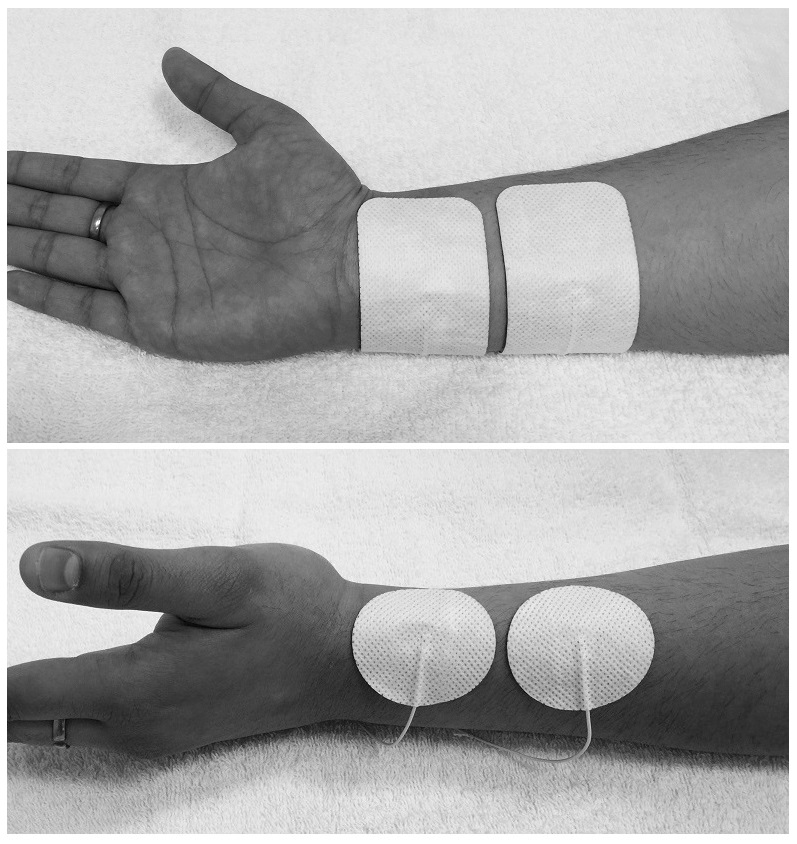

Supplement: Supplementary file 1 — (A) Placement of the rectangular electrodes overlapping the stimulation sites of the median and ulnar nerves. (B) Placement of the circular electrodes over the stimulation site of the radial nerve. (TIFF 846 kb) [file 12984_2017_323_MOESM1_ESM.tif]

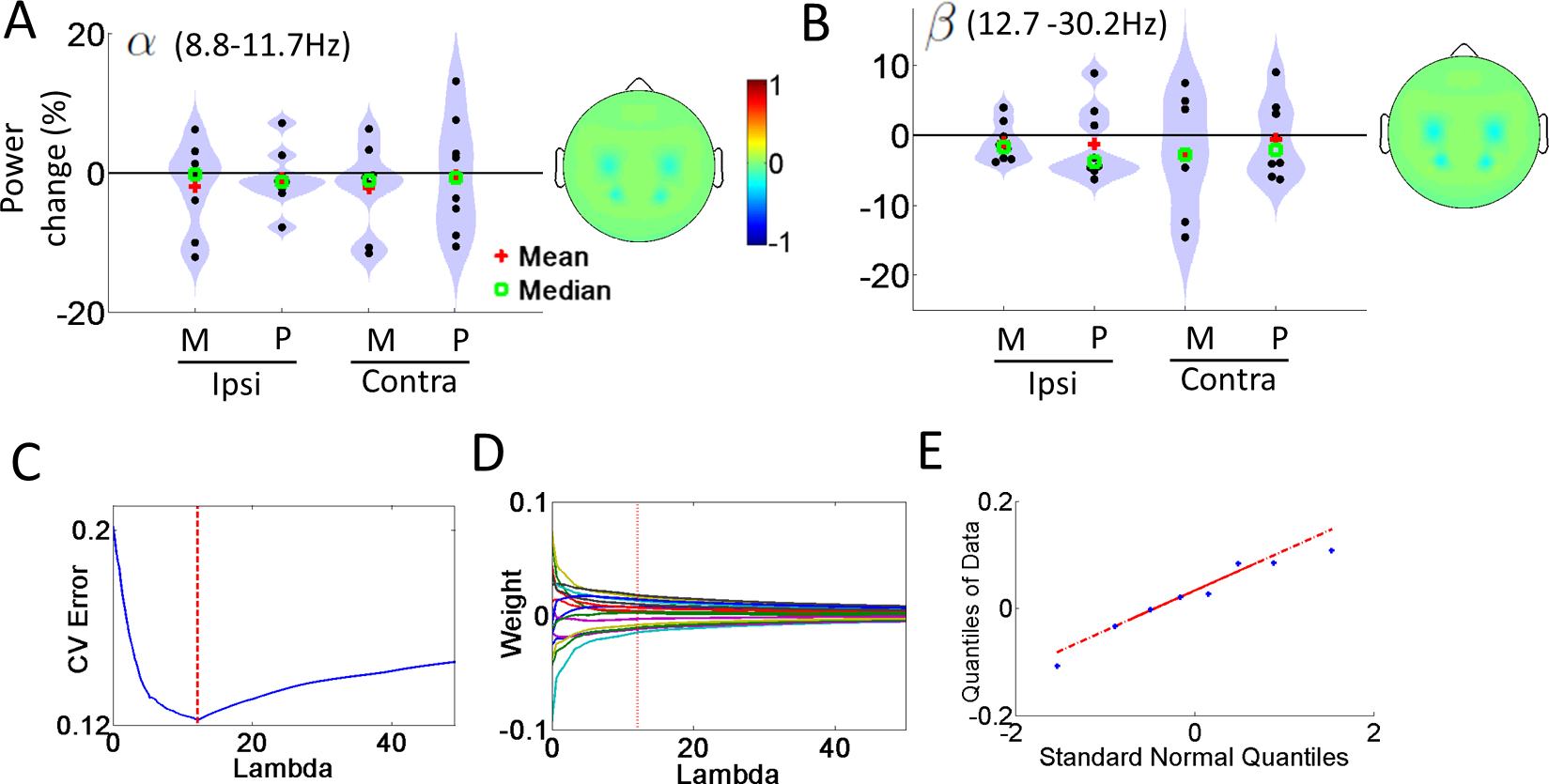

Supplement: Supplementary file 2 — Distribution of percentage change in mean resting state EEG power across the eight subjects, pre to post intervention, within the A) alpha frequency band and B) beta frequency band with head plots depicting 1/coefficient of variation (mean/standard deviation) of group level percentage changes. There were no significant differences. C) Result from the leave-one-out cross validation (CV) procedure to find the optimal ridge parameter (lambda) that produced the lowest CV error given by the vertical dotted red line. D) Ridge trace plotting the coefficient weights of the multivariate ridge model for various values of the ridge parameter with the optimal lambda indicated by the dotted red line. E) Quantile plots from the weighted residuals of the Huber robust regression. M: electrodes over Motor cortex; P: electrodes over Parietal cortex. (TIFF 362 kb) [file 12984_2017_323_MOESM2_ESM.tif]
